# The ABCD and ABCD2 Scores and the Risk of Stroke following a TIA: A Narrative Review

**DOI:** 10.5402/2011/518621

**Published:** 2011-07-21

**Authors:** Archit Bhatt, Vishal Jani

**Affiliations:** ^1^Spectrum Health, Grand Rapids, MI 49503, USA; ^2^Michigan State University College of Human Medicine, Grand Rapids, MI 49503, USA; ^3^Department of Neurology, Michigan State University, East Lansing, MI 48824-1046, USA

## Abstract

The California, ABCD, and ABCD2 risk scores (ABCD system) were developed to help stratify short-term stroke risk in patients with TIA (transient ischemic attack). Beyond this scope, the ABCD system has been extensively used to study other prognostic information such as DWI (diffusion-weighted imaging) abnormalities, large artery stenosis, atrial fibrillation and its diagnostic accuracy in TIA patients, which are independent predictors of subsequent stroke in TIA patients. Our comprehensive paper suggested that all scores have and equivalent prognostic value in predicting short-term risk of stroke; however, the ABCD2 score is being predominantly used at most centers. The majority of studies have shown that more than half of the strokes in the first 90 days, occur in the first 7 days. The majority of patients studied were predominantly classified to have a higher ABCD/ABCD2 > 3 scores and were particularly at a higher short-term risk of stroke or TIA and other vascular events. However, patients with low risk ABCD2 score < 4 may have high-risk prognostic indicators, such as diffusion weighted imaging (DWI) abnormalities, large artery atherosclerosis (LAA), and atrial fibrillation (AF). The prognostic value of these scores improved if used in conjunction with clinical information, vascular imaging data, and brain imaging data. Before more data become available, the diagnostic value of these scores, its applicability in triaging patients, and its use in evaluating long-term prognosis are rather secondary; thus, indicating that the primary significance of these scores is for short-term prognostic purposes.

## 1. Introduction

Annually, approximately 240,000 TIAs are diagnosed in the United States [1]. TIAs admissions represent approximately 0.3% of ED (Emergency Department) visits [2], and about 23% of strokes are preceded by a history of TIA [3]. Recent studies have suggested that early care and rapid ED initiated treatment and diagnostic protocols within 24 hours can reduce post-TIA stroke rates significantly [4–6]. 

The short-term risk of stroke after a TIA is substantially higher than previously thought and significantly higher than the short-term risk of recurrent stroke. After a stroke, the 30-day risk of stroke is estimated to be 1.5% (CI 0.6–2.5), [7], whereas the risk of stroke following TIA was 3.1% (95% CI 2.0–4.1) after 2 days and 5.2% (3.9–6.5) after 7 days [8]. Over recent years, three clinical prediction rules (ABCD, California, and ABCD2) (Table 1) have been developed with a purpose of predicting short-term risk of stroke [9, 10]. Although, initially developed for prognostic purposes, some studies have assessed the diagnostic value of these rules for TIA [11, 12]. These scoring systems are based on simple clinical information that are readily obtained at a first clinical encounter, that is, age, duration, and type of symptoms, and presence of elevated blood pressure or diabetes. The rules do not incorporate other variables known to also predict short-term risk of stroke such as Diffusion Weighted Imaging abnormalities (DWI) [13], large artery stenosis [14], and atrial fibrillation [15]. 

The principal objectives of our review are to summarize the prognostic value of these scores to predict short term post-TIA stroke risk, to review the diagnostic capability or accuracy of these scores in identifying TIA patients, to explore the relationship between these scores and other prognostic indicators of stroke risk in TIA patients which include, presence of diffusion weighted abnormalities, large artery stenosis, and atrial fibrillation, and finally, to discuss the potential role of these risk scores in triaging patients with TIA in the ED.

## 2. The Prognostic Value of ABCD and ABCD2 Scores in Predicting Short-Term Risk of Stroke after a TIA

The California Rule and the ABCD score (Table 1) were initially developed to predict short-term risk (2 days, 7 days, 30 days, and 90 days) of stroke in TIA patients. They were subsequently combined to create a new rule, called the ABCD2 score (Table 1), with the goal of creating a more comprehensive value [16]. These rules include presence of stroke risk factors like diabetes and hypertension, symptoms—unilateral weakness and speech impairment, and duration of these symptoms, which have shown to have an independent prognostic value because they improve the diagnosis of TIA from non-TIA disorders [17]. 

## 3. Short-Term Risk Prediction with ABCD and ABCD2 Score

We identified studies specifically evaluating ABCD score and reporting 2-day stroke risk [16], 7-day risk of stroke, [10, 16, 18–25]; 30-day stroke risk [20, 21, 23], and 90-day stroke risk [16, 24]. Patients were dichotomized to (ABCD < or = 3, low risk versus ABCD > 3, intermediate to high risk). In patients with low risk ABCD score, the 2-day, 7-day, 30-day, and 90-day risk ranges were 1.2%, 0–5.9%, 0–5.4%, and 0–3.2%, respectively. Whereas in patients with intermediate to high risk score, the 2-day, 7-day, 30-day, and 90-Day risks were 4.9–7.9%, 4.2–15.9%, 6.9–17.6%, and 11.3–18.9%, respectively.

Few studies [11, 16, 21, 23, 26] have evaluated the ABCD2 score in predicting short-term stroke risk. Studies [16] have estimated 2-day risk of stroke, 7-day risk of stroke [16, 21, 23, 27], 30-day risk of stroke [21, 23], and 90-day risk of stroke [11, 23]. The short-term stroke risks for low risk patients with ABCD2 score of 3 or less, at 2 days, 7 days, 30 days, and 90 days ranged between 0.8% to 2.5%, 1.2% to 5.9%, 1.2–5.9%, and 5.3 to 6.6%, respectively. In intermediate to high-risk patients (ABCD2 > 3), short-term stroke risks at 2 days, 7 days, 30 days and 90 days were 4.2 to 8.9, 5.9 to 14.7, and 9.6 to 26.9 percent respectively. 

### 3.1. Accuracy of These Scores

The above results suggest that there is a wide variability in use of scores and stroke risk among studies. This questions the generalizability, accuracy, and equivalency of these scores. In general, the discriminatory capability of a prognostic score is measured by the area under the curve (AUC) of a ROC curve where sensitivity is plotted against specificity [28, 29]. The larger the area, the better the diagnostic test with a maximum score of 1.0, corresponding to 100% sensitivity and 100% specificity. If the area is 0.5, then you have a test, which has effectively 50% sensitivity and 50% specificity. The closer the area is to 1.0, the better the test is, and the closer the area is to 0.5, the worse the test is. Values less than  .75 have fair accuracy,  .75–.92 have good accuracy,  .92–.97 have very good accuracy and  .97–1.00 has excellent accuracy. Three pooled analyses have shown that ABCD2 scores are similar as compared to ABCD, and California scores in predicting short-term risk of stroke after a TIA. In a pooled analysis of 6 cohorts [16, 30], the ABCD2, ABCD and California scores have similar accuracy predicted for 2-, 7-, or 90-day stroke risk (AUROC curve  .62–.83 versus  .62–.81 versus  .60–.79). In a pooled estimate of 13 cohorts, which included 5938 subjects and 332 strokes, the AUC for ABCD and ABCD2 scores, respectively, were  .70 (.66–.73) and  .70 (.66–.74) for 7-day risk of stroke risk and  .68 (.65–.71) and  .69 (.66–.72) for 90-day risk [31]. A recent systematic review of 20 cohorts showed that the ABCD and ABCD2 scores were similar in their prognostic value in predicting 7-day risk of post-TIA stroke—pooled AUC for ABCD was 0.72 (.66–.78) and pooled AUC for ABCD2 0.72 (0.63–0.80). The predictive value in the same review had significant variations between studies (*P* < .001). Also, independent face-to-face validations had a higher predictive value than retrospective data [32]. 

### 3.2. Distribution of Scores

Studies evaluating ABCD and ABCD2 scores in TIA patients have suggested that most patients are categorized in medium to high-risk category and the majority of strokes occur in medium to high-risk category. For studies (ABCD) reporting 7-day risk of stroke (Table 2), patients mainly (range 84.97 percent to 54.1 percent) fall into the intermediate or high-risk category (ABCD > 3). Nine out of the 10 studies (except Purroy et al. [19]) showed that majority of subsequent strokes (range, 81.8% to 100%) occur in patients with ABCD score > 3, within 7 days. Five [10, 20, 21, 24, 33] out of ten studies reporting 7-day risk and one [20] out of four studies reporting 30-day risk did not have any patients with subsequent strokes whose ABCD scores were less than 4. In four studies [20, 21, 23, 33] that reported 7-day and 30-day risk of stroke, 50 to 87.8% of strokes occurred in the first seven days. 

Similar distribution patterns were observed in studies reporting ABCD2 score (Table 3). For 6 studies (ABCD2) reporting 7-day risk of stroke (Table 2), patients mainly (range 84.97 percent to 62 percent) fall into the intermediate or high-risk category (ABCD > 3). However, the majority of strokes in the first seven days, which reported in these studies, fell into the intermediate to high-risk category (range, 86.7% to 94.5%). The majority of studies (Table 4) have shown that more than half of the strokes in the first 90 days occur in the first 7 days (range 29% to 100%), and the rates are even higher in patients classified in ABCD/ABCD2 > 3, further indicating that aggressive intervention and work up is necessary immediately after a TIA to attenuate short-term risk of stroke.

### 3.3. High-Risk Patients with ABCD and ABCD2 (Score > 3 or 4)

ABCD or ABCD2 scores (>4) = have a substantially higher early 7-day risk of stroke, which ranges between 5.6 and 23.8 percent [16, 20, 21, 24, 33–35]. Studies have shown that [21, 33] the risk of post-TIA stroke within the first 30 days was incremental with a higher ABCD score. Two studies [33, 35] have shown that majority of strokes occurred in these high risk categories (scores > 4). First study showed that 80% strokes were in the high ABCD2 > 4 category [35], and in the second study [33] showed that all subsequent strokes (*n* = 4, 8.3%) within the first 7 days after a TIA had an ABCD score > 4. A study by Calvet [24] showed that 5 out of 57 patients (8.8%) with score 5 and 6 on ABCD had strokes within 7 days. Fothergill et al. [23] showed that the risk of stroke at 7 days and 30 days according to ABCD2 scores for patients with score >4 was 19.2% and 20.7%, respectively. In the largest validation study involving [16] 4,800 TIA cases it was shown that the ABCD2 score was highly predictive of subsequent stroke risk. Twenty-one percent of these cases were classified as high risk based on the fact that they had a score of 6 or 7, which was associated with a very high 2-day stroke risk of 8.1%. Forty-five percent of cases were classified as moderate risk on the basis of a score of 4 or 5, which was associated with a 2-day stroke risk of 4.1%. Finally, 34% of cases were classified as low risk (score ≤ 3, 2-day stroke risk = 1.0%). Whereas in oxford validation cohorts [16], the 2-day risk was 8.4% (27/321) and 7-day risk was 13.7% (44/321). Similarly other ABCD (> 4) based studies [19, 20], reported 7-day risks at 7/123 (5.69%) and 8/114 (7.02%), respectively. All these studies consistently indicate that patients classified with ABCD or ABCD2 scores higher than 4 have a substantially higher risk of early stroke. 

### 3.4. Low Risk ABCD and ABCD2 Scores

Low risk scores may have substantial stroke risk [18, 19, 23]. This may be due to the fact that they may have other high-risk cause of stroke such as high signal on DWI, LAA, or atrial fibrillation. A population-based retrospective analysis [23] of ABCD and ABCD2 showed that 25% strokes (9/36) occurred in patients with ABCD2 score < or = 4. The risk of stroke in low risk patients (< 4) was 5.9%. Patients [18] with a score <4 still have a substantial probability of having a high-risk cause of cerebral ischemia or radiographic evidence of acute infarction despite transient symptoms independent of ABCD score. In a study of 345 patients [19], in low risk (ABCD < 4) category, 5.8% had strokes within 7 days. Large artery atherosclerosis and not the ABCD score was the only independent predictor for stroke. 

On the other hand, a prospective observational study [36] of 637 patient, showed that there was no relationship between ABCD2 score at the presentation and subsequent stroke risk after TIA (*P* = .48). At 7 days, risks of stroke were 1.1%, 0.3%, and 2.7% in low, intermediate and high risk groups. At 30 days, the risk of stroke were 2.1, 2.1, and 3.6 percent for low, intermediate, and high risk groups respectively. 

### 3.5. Usefulness in Predicting Long-Term Risk and Overall Vascular Risk

Few studies have evaluated long-term risk of stroke. A study reported that 1-year risk [23] of stroke in patients with TIA with ABCD score > 4 was 31 percent and for < 4 was 16 percent. Another study (Harrison 2010) showed that ABCD or ABCD2 Scores of >2 predicted raised stroke risks at 1, 5, and 10 years. AUCs were 0.619 (95% CI 0.571–0.668) and 0.630 (95% CI 0.582–0.677) for the ABCD and ABCD2 scores, indicating fair accuracy. Similarly, Yang et al. in 2010 showed that ABCD2 score > 4 was found to be an independent risk factor for long-term risk of stroke (up to 3 years) (HR, 2.27; 95% CI, 1.36 to 3.80) and for death (hazard ratio, 1.68; 95% CI, 0.99 to 2.85). A study further indicated that ABCD2 score > 3 was significantly associated with the combined endpoint of cerebral or cardiovascular ischemic events, including MI, stroke, and revascularization, and death of vascular or unknown cause (hazard ratio (HR) 4.01, 95% confidence interval (CI) 1.21 to 13.27). These studies indicate that ABCD/ABCD2 scores may help with intermediate or long-term prognosis in predicting not only stroke but also other vascular events.

## 4. California, ABCD, and ABCD2 Scores, Clinical Features, and Diffusion-Weighted Imaging

In patients with TIA, symptom duration > or = 60 minutes, dysphasia, dysarthria, unilateral weakness, atrial fibrillation, and ipsilateral carotid stenosis were independently associated with presence of diffusion-weighted imaging abnormalities. However, age, sex, hypertension, and diabetes were not associated with presence of DWI lesions [13]. 

Several studies have tried to determine the association of ABCD or ABCD2 score and Diffusion Weighted Imaging [18, 43–45]. Two studies have found association [43, 44] of DWI abnormalities with increasing scores and two studies [18, 45] have found poor correlation between the scores and the presence of DWI abnormalities. The presence of DWI lesions in patients with TIA can provide useful prognostic insights. As mentioned previously, many TIA patients have DWI abnormalities [13, 46, 47], and these changes are associated with more definitive TIA symptoms such as unilateral weakness, speech disturbance, and vascular risk factors such as large artery atherosclerosis and atrial fibrillation [44]. In a study of 200 TIA patients who underwent brain imaging 3 or more days after the event, higher scores of California and ABCD rules (which indicates higher short-term stroke risk) were associated with positive DWI lesions [44]. Recently, two prospective studies by Purroy et al. [45, 48] showed that high ABCD, ABCD2, and California scores were not associated with DWI abnormalities, but rather clinical symptoms like facial palsy, motor weakness, and large artery atherosclerosis were associated with a positive DWI lesions. Another study by Cucchiara et al. [18] revealed that presence of unilateral weakness rather than ABCD score was predictive of DWI but the increasing ABCD score correlated poorly with presence of DWI lesions (*P* for trend =.24). Sixty percent of DWI (+) patients were high risk compared to 8.7% DWI (−) patients (OR, 15.8, 95% CI, 3.7 TO 67.5). Even after adjusting for ABCD score, the presence of DWI+ lesion remained a significant predictor of high-risk category. Although unilateral weakness and speech disturbance predicted DWI+ lesions, even in absence of these symptoms 15% of patients were high risk (> or = 50% stenosis or cardioembolic stroke) or had DWI + (8%) lesions. Also frequency of high-risk patients increased with an increasing ABCD score but the increase was not statistically significant (*P* for trend =  .11). In a different study by Calvet et al. [43], which included 339 patients of TIA who underwent DWI and were followed up for 3 months, diffusion-weighted imaging was positive in 40% patients. Factors predictive of DWI lesions were unilateral weakness, TIA duration >/=60 minutes, ABCD2 score > 5, large artery atherosclerosis, and atrial fibrillation. In the same study, ABCD2 score, large artery atherosclerosis, and positive DWI findings were independently associated with an increased 7-day and 3-month risk of stroke. However, atrial fibrillation was not significantly associated with short-term risk of stroke. In another study by Calvet et al. [24] which included 203 consecutive patients with TIA showed that ABCD score of  ≥ 5 (HR = 5.0; 1.0–25.8; *P* = .06) and presence of DWI abnormalities (HR = 10.3; 1.2–86.7; *P* = .03) were independently associated with 90-day risk. Also presence of DWI abnormalities (*P* = .001) and ABCD score were also associated with a 7-day risk (*P* = .005). There were 5 strokes within seven days all had positive diffusion weighted abnormalities and an ABCD score of more than 3. At 90 days, there were 7 strokes of which all had a score of more than 3, and 6/7 patients had positive DWI abnormalities. Among the components, age, mean duration of symptoms, and dysphasia were strongly associated with positive DWI in the same study, all of which are components of the ABCD score. Several studies have shown that positive DWI is associated with a short-term risk of stroke at 90 days [41, 49]. Redgrave et al. suggested that atrial fibrillation (OR 5.87, 95% CI, 1.95-17.67, *P* = .002) was strongly associated with DWI positive lesions [44]. 

In a study by Asimos et al. [25], a total of 1168 patients had MRI performed within 24 hours, of which 331 (28%) were DWI positive, including 33 patients with ABCD2 < or = 3. In the same population when information of a low ABCD2 score (<4) and a negative early DWMRI was combined, it yielded excellent sensitivity (100%, 95% CI 34 to 100) for identifying low-risk patients. A study by Sciolla [20], which involved 274 patients, included a modified ABCD-I score in which I stands for imaging to incorporating CT findings. The ABCD score was predictive of stroke risk (7 day  .018, 30 day  .0017), but ABCD1-I improved predictive value of stroke risk (7 day *P* = .0043, 30 DAY.0003). Similarly, adding imaging to the ABCD2 score [40] increased the area under the curve (predictive accuracy) from 0.66 (95% CI, 0.57 to 0.76) to 0.81 (95% CI, 0.74 to 0.88; *P* = .003). These data indicate that individual clinical features and presence of high-signal on DWI are both critical in adjunct to use of ABCD/ABCD2 scores in predicting short-term post-TIA stroke risk. 

## 5. ABCD and ABCD2 Scores with Large Artery Atherosclerosis and Atrial Fibrillation

Early surgery for extracranial carotid disease within 1-2 weeks is prudent in patients with TIA [50, 51]. Delaying surgery for symptomatic carotid stenosis (70–99%) for >12 weeks prevented only eight strokes per 1000 CEAs (Carotid Endarterectomy), compared to more than 180 strokes prevented, per 1000 CEAs, when CEA was performed within two weeks of last cerebrovascular event [52]. A 90-day risk of stroke with ipsilateral carotid diseases is 20.1% [53]. In spite of increasing use of ABCD2 score for stratifying TIA patients, few studies have analyzed the relationship of the ABCD2 score and presence of ECS and ICS stenotic lesions on vascular imaging. All population-based studies reporting early risk of recurrent stroke according to subtype were pooled, and 1709 patients with stroke were included with 30 recurrent strokes at 7 days, 72 at 30 days, and 113 at 3 months. At each time interval, the risk of stroke recurrence was highest in patents with large artery atherosclerosis, mainly carotid stenoocclusive disease and was lowest in patients with small vessel disease. Cardioembolic strokes fell into the intermediate category [14]. Among earlier studies, a study by Tsivgoulis et al. [21] showed a higher prevalence of ipsilateral carotid stenosis in patients with score of 5 or 6 compared to patients with ABCD < or = 4. Among them, a recent analysis by Quinn et al. [12] of 1877 TIA clinic patients suggested that a high ABCD2 was associated with presence of carotid stenosis (*P* < .  001). In another study by Koton and Rothwell [35], which included 285 patients, the ABCD and ABCD2 scores were highly predictive of stroke at 7 days (*P* < .0001). The study found no convincing relationship between either score or the prevalence of 50% or greater carotid stenosis (ABCD  .27, ABCD2  .29) or Atrial fibrillation (ABCD2, *P* = .86, ABCD, *P* = .90). Six patients in that study with AF or symptomatic stenosis who had a stroke within 7 days of their TIA had an ABCD2 score of > or = 4. 

In a study of 117 patients [18], the ABCD score had some predictive value in identifying high risk patients (with carotid stenosis and hyper-intense DWI lesions); however, patients with a score < 4 still had a significant probability of having a high-risk cause (atrial fibrillation or carotid stenosis) of cerebral ischemia or radiographic evidence of acute infarction despite transient symptoms. 

In a study by Calvet et al. [43], which included 343 patients with TIA, it was found that large artery atherosclerosis (LAA) was an independent predictor of stroke at 3 months (HR = 4.9, CI 1.4–16.9, *P* = .006) post-TIA. However, in the same study the absolute stroke risk did not differ at 7 days post-TIA (*P* = .18) in patients with LAA compared to who did not have LAA. In a study of 345 patients by Purroy et al., it was found that ABCD score was not a predictor of stroke; however, the only predictor was large-artery occlusive disease (HR 5.88, 95% CI, 2.17 to 15.89; *P* < .001) [19]. 

Intracranial and extracranial occlusion also is an independent risk factor of recurrent stroke [41, 54]. A French study showed that ABCD2 > 3 on admission of all TIA patients who predicted intracranial narrowing or occlusion was 2.29 (95% confidence interval [CI], 1.15–4.56; *P* = .02) [55]. A recent study [56] evaluating ABCD2 score evaluating 276 patients by Schrock et al. suggested that ABCD2 score > or = 4 was associated with increased likelihood of carotid stenosis (OR 3.78, 1.03–13.78, *P* < .05). Carotid stenosis and not the ABCD2 score predicted 90-day stroke risk (HR = 2.56; 95% CI, 1.27 to 5.15, *P* = .003) [42]. A study [57] showed that one in five ABCD(2) score < 4 had high-risk disease requiring urgent treatment decision-making like carotid stenosis >50%, intracranial stenosis, atrial fibrillation, or other cardioembolic source. 

Atrial fibrillation is an independent risk factor for stroke [58]. Overall limited data is available predicting atrial fibrillation in relation to ABCD and ABCD2 scores. The researchers [33] also found that a history of atrial fibrillation was not of any predictive value. Twelve (12%) patients had history of atrial fibrillation, or atrial fibrillation was detected by EKG. Only one patient had a stroke within 7 days. His ABCD score was high. A study by Quinn et al. [12] did not suggest any association of atrial fibrillation (*P* = .097) and ABCD2 score. These data make it clear that the presence of large artery atherosclerosis and atrial fibrillation cannot be completely predicted by ABCD/ABCD2 score, and their detection may be a key in short-term and long-term risk reduction.

## 6. ABCD and ABCD2 Scores with the Presence of Noncerebrovascular Diagnosis

Although most studies have focused on prognostic value of the ABCD and ABCD2 scores, few studies have evaluated the role for ABCD2 score and its diagnostic value with cerebrovascular and noncerebrovascular diagnosis [11, 12, 59].  In an earlier study [11] out of the 1707 patients with TIA, 713 patients with questionable TIA were reviewed by the expert neurologist and 642 (90%) were adjudicated as true TIAs. ABCD2 scores were higher in those judged to have a true TIA compared to others (*P* = .0001). In the same study, the 90-day stroke risk increased with increasing ABCD2 score (*P* ≤ .0001) in patients with “true TIAs”. However, this trend did not hold in patients not adjudicated as TIAs (*P* = .73). In a prospective audit of 75 patients [59], 43 (57.3%) patients had a confirmed diagnosis of stroke or TIA. The median ABCD score for stroke or TIA diagnosis was 4 and for a noncerebrovascular diagnosis was 2. The sensitivity of ABCD2 score of greater than 2 for stroke or TIA diagnosis was 88% with an odds ratio of 16.7 (confidence interval = 5.1 to 44.2). However, it was important to note that 20% of patients with ABCD score less than 3 had a final diagnosis of TIA, and 33% of patients with ABCD score 3 or more had a final diagnosis consistent with a noncerebrovascular diagnosis. A large retrospective data base in West Glasgow Stroke Registry (*N* = 3705) of patients by Quinn et al. [12] showed that higher ABCD2 score was associated with cerebrovascular diagnosis (*P* < .001) and a positive predicitive value of a low ABCD2 score (0 or 1) was  .81 for noncerebrovascular diagnosis. Median ABCD2 score in cerebrovascular disease was 4(3–5) and in noncerebrovascular disease was 2(1–4). Analysis of ROC curve for use of ABCD2 in diagnosis of noncerebrovascular events showed modest sensitivity, (.745, 95% CI,.729 to  .761) at the cost of specificity. In a Stanford cohort [60] of 152 TIA patients referred to a rapid access TIA clinic, one patient had a stroke at the end of 7 days. The patient was in a low-risk ABCD category and a higher-risk ABCD2 category. However, based on the above data, assigning a cut-off of ABCD2 score for prediction of a cerebrovascular or noncerebrovascular diagnosis would be difficult. These data signify that noncerebrovascular diagnoses is common in TIA patients, and justifiably, higher ABCD2 score may predict a vascular diagnosis. But patients with low risk scores can have vascular diagnosis and thus, the diagnostic yield of the ABCD/ABCD2 scores is limited.

## 7. Current Role and Limitations of ABCD and ABCD2 Scores

In a pooled analysis, stroke risk in 10 126 TIA patients was 5.2% (95% CI 3.9–6.5) at 7 days. The lowest risks were seen in studies of emergency treatment in specialist stroke services (0.9% (95% CI 0.0–1.9), four studies) and the highest risks in population-based studies without urgent treatment (11.0% (8.6–13.5), three studies) [8]. The SOS-TIA and EXPRESS studies have suggested that expeditious evaluation in patients with TIA in the emergency department is critical to reduce short-term risk of stroke [5, 6]. 

There are few limitations to the ABCD system. Firstly, it does not allow recognition of stroke subtype such as LAA, cardioembolic (e.g., atrial fibrillation), and lacunar, partly because vascular imaging and cardiac data is not incorporated in the ABCD system. Moreover, diffusion-weighted imaging and clinical features have shown to be an independent predictors of short-term stroke risk independent of the ABCD scoring system. Therefore, even in low risk patients clinically significant etiologic factors compound the stroke risk. There are at least two studies, which have shown that even low risk patients had significant high stroke risk. Cucchiara et al. [18] found that predictive value of ABCD score is not optimal, and patients with ABCD score of 0–3 still had clinically significant probability (10–20%) of a stroke risk at 90 days or a high risk because of cerebral ischemia warranting intervention. The same study found low risk of deaths 2 and 2 strokes at 90-day. A similar prospective observational study [36] of 637 patients showed that there were a total 15 strokes within 90 days followup. There was no relationship between ABCD2 score at the presentation and subsequent stroke risk after TIA (*P* = .48). At 7 days, risk of stroke were 1.1%,  .3%, and 2.7% in low, intermediate, and high risk groups. At 30 days, the risks of stroke were 2.1, 2.1, and 3.6 percent for low, intermediate, and high risk groups respectively. In third study by Cucchiara et al., [38], 167 TIA patients enrolled ABCD2 score was associated with high-risk status (carotid stenosis, atrial fibrillation) (*P* = .015) but not associated with positive diffusion weighted abnormalities (*P* = .81). 

There is paucity of data where ABCD system predicts the TIA territory. The system does not allow differentiation of anterior versus posterior circulation TIAs. Short-term risk of stroke also depends on the vascular territory of the event. A systematic review of 37 published cohort studies showed that the risk of stroke after TIA or minor stroke was higher with carotid territory strokes as compared to vertebrobasilar events [61]. In addition,  monocular events (amaurosis fugax), for instance, have a lower subsequent risk of stroke [62]. 

Further, most research evaluating or validating ABCD and ABCD2 scores has been done in a retrospective fashion. Therefore, this limits optimal value of the data. Also, most studies evaluated patients who are hospitalized thus limiting the access to outcome data for low risk patients that are typically discharged. Thus validation is needed especially in patients who are discharged from the ED. This can only be obtained by creating prospective population-based community wide registries, rather than hospital based or ED-based registries. 

Assigning an absolute cut-off of an ABCD/ABCD2 score would be difficult; however, the ABCD2 score may be used to “triage” referrals to TIA services, which would facilitate prudent utilization of resources. Widespread use of ABCD system in clinical practice has incorporated into the guidelines [63], which state that it is reasonable to hospitalize patients with TIA if they present within 72 hours and have an ABCD2 score > or = 3, indicating high risk of early recurrence, or the evaluation cannot be rapidly completed on an outpatient basis. In a study by Sciella and Helis [20], the mean ABCD score for admitted patients was 4.23 and for discharged patients was 3.96 (*P* = .04). In a large Austrian cohort, ABCD2 score predicted neurological worsening (more than 2-point worsening in NIHSS) in patients with TIA or minor stroke [64].

Practices regarding admission after TIA vary widely, with admission rate of around 50% which has not increased over the past decade [2]. Recent studies [5, 6] have shown that immediate access to TIA clinics can substantially reduce the short-term risk of stroke by 80%. A cost utility analysis showed that 24-hour hospitalization for TIA could be cost effective only if patients had higher likelihood that will receive thrombolysis—that is, if they develop an acute ischemic stroke [65]. Calvet et al. [24] 2007 showed that in five patients who had strokes while in hospital, 2 received intra-arterial tPA. Also the same study showed that 30% of patients admitted with TIA had additional treatments which included carotid revascularization, anticoagulation and acute stroke intervention. In a study by Josephson et al. [66], all patients diagnosed with TIA of 16 hospitals of Kaiser-Permanente Medical Care Plan and the admission weakly correlated with ABCD2 score *R* ^2^ = .036; 10% at low 2-day risk of stroke admitted versus 20.3% at high risk. Variables associated with decision to admit were prior TIA, speech impairment, weakness, gait disturbance, history of atrial fibrillation, and symptoms on arrival to ED. Using data from 1176 patients with a definite or possible transient ischemic attack or minor stroke included in the SOS-TIA registry [57] (January 2003 to June 2007), which studied the usefulness of the conventional ABCD(2) score cutoff for urgent admission to a stroke unit defined as presence of symptomatic internal carotid artery stenosis >/=50%, symptomatic intracranial artery stenosis >/=50%, or major cardiac source of embolism. Although independent prognostic values of ABCD2 scores have been established, the prognostic value of other independent factors such as presence of large artery atherosclerosis, atrial fibrillation, and diffusion-weighted imaging cannot be underestimated with DWI having an additional diagnostic value. The combination of clinical, radiological, and vascular information can be useful in making the prognosis inclusive in order to prevent stroke recurrence. The paper by Bray [22] prompted the authors to form a TIA pathway based on the ABCD score. Education sessions were provided to ED medical staff and 87 patients were audited. Use of TIA pathway was found only in 23% patient records (*n* = 20). There was an increase in number of inpatient CT scans (*P* = .007). ABCD system poorly correlated with triage. A study conducted by Min Lou et al. analyzed prospectively collected data from 151 consecutive TIA patients admitted [67]. Eleven percent had abnormalities on DWI imaging, of which 57% had a score of < or = 3. The study found that there was no difference in patients getting in hospital intervention (anticoagulation or revascularization) among low risk versus high-risk patients. Ten percent of low risk patients got intervention and 10 percent of medium to high-risk patients got intervention (*P* = .8). In a periodic review of Greater Cincinnati/Northern Kentucky Stroke Study [68], of the 603 TIA patients, 482 patients were admitted. Seventy-nine percent (*n* = 439) were considered moderate to high-risk as per ABCD2 score (>3). Factors associated with admission to the hospital were younger age (*P* = .045), higher NIHSS (*P* < .001), and unilateral weakness (*P* = .002). Moderate to high ABCD2 score was associated with admission to the hospital from the ED (*P* = .003). However, the ABCD2 score categorized four of every 5 patients as moderate to high-risk, which may limits the utility in triaging. We believe that there is sufficient data indicating expeditious evaluations of patients with high (>3) ABCD2 scores; however, data are insufficient as to how patients with lower ABCD2<3 should be triaged.

## 8. Conclusion

California and ABCD/ABCD2 scores have equivalent accuracy in predicting short-term risk of stroke. High risk and even low risk patients may have vascular stenosis or cardioembolic source. Larger studies are therefore needed to address this void between prognostic information (ABCD system), etiologic factors (large artery atherosclerosis, atrial fibrillation), and presence of DWI abnormalities. In summary, the ABCD system facilitates prognosis of TIA cases in the acute setting, especially those at moderate or high risk of stroke in whom urgent evaluation and intervention is justified. Conversely, patients with a low ABCD2 score are at low risk, in part because many of them are likely not TIA cases. Low risk TIA cases may not require urgent evaluation, although such a clinical strategy has yet to be formally tested, as many have high-risk causes of subsequent stroke.

## Figures and Tables

**Table 1:** Risk scoring systems.

Clinical prediction rule	Components	Score
	Age ≥ 60	1
	Diabetes	1
	Unilateral weakness	1
California rule [9]	Speech impairment	1
	Symptom duration >10 minutes	1
	Total	5

	Age ≥ 60	1
	Elevated blood Pressure	1
	Systolic ≥140 mm Hg	
	Diastolic ≥ 90 mm Hg	
	Unilateral weakness	2
ABCD rule [10]	Speech impairment	1
	Symptom duration	
	≥ 60 minutes	2
	10–59 minutes	1
	< 10 minutes	0
	Total possible score	6

	Age ≥ 60	1
	Elevated blood pressure	1
	Systolic ≥140 mm Hg	
	Diastolic ≥90 mm Hg	
	Diabetes	1
	Unilateral weakness	2
ABCD2 rule [17]	Speech impairment	1
	Symptom duration	
	≥ 60 minutes	2
	10–59 minutes	1
	< 10 minutes	0
	Total possible score	7

**Table 2:** 2-day, 7-day, and 90-day estimates of the RISK OF STROKE by low and high ABCD.

Risk category	ABCD 3 or less (*N*)		ABCD 4 or more (*N*)	
	Low risk score (*N*)		High risk score (*N*)	
	*N*	2 day risk % (*N*)	*N*	2 day risk % (*N*)

California, [16] 2005 (*N* = 3738)	562	1.2 (7)	3176	4.9 (157)
Oxford, [16] 2005 (*N* = 649)	247	1.2 (3)	402	7.9 (32)

	*N*	7-day risk % (*N*)	*N*	7-day risk % (*N*)

Rothwell et al., [10] 2005 (*N* = 188)	62	0 (0)	126	15.9 (20)
Tsivgoulis et al., [21] 2006 (*N* = 226)	97	(0)	129	13.2 (17)
Purroy et al., [19] 2007 (*N* = 345)	119	5.8 (7)	226	4.4 (10)
Bray et al., [22] 2008 (*N* = 98)	35	0 (0)	63	6.4 (4)
Sciolla and Melis, [20] 2008 (*N* = 274)	58	(0)	216	4.2 (9)
California, [37] 2005 (*N* = 3738)	562	1.5 (8)	3176	6.6 (211)
Oxford, [37] 2005 (*N* = 649)	247	1.2 (3)	402	9.9 (40)
Calvet et al., [24] 2007 (*N* = 203)	84	0 (0)	99	5.1 (5)
Kontonand Rothwell, [35] 2007(*N* = 278)	96	NA	182	NA
Fothergill, [23] 2009 (*N* = 284)	74	5.9 (4)	210	15.2 (32)

	*N*	30-day risk % (*N*)	*N*	30-day risk % (*N*)

Tsivgoulis, [21] 2006 (*N* = 226)	(2.06) 97	(2)	129	15.5 (20)
Bray et al., [22] 2008 (*N* = 98)	35	2.9 (1)	63	12.7 (8)
Sciolla and Melis, [20] 2008 (*N* = 274)	58	(0)	216	6.9 (15)
Fothergill, [23] 2009 (*N* = 284)	74	5.4 (4)	210	17.6 (37)

	*N*	90-day risk % (*N*)	*N*	90-day risk % (*N*)

California, [37] 2005 (*N* = 3738)	562	3.2 (18)	3176	11.3 (360)
Oxford, [37] 2005 (*N* = 649)	247	2.4 (6)	402	18.9 (76)
Calvet et al., [24] 2007 (*N* = 203)	84	0 (0)	99	7.2 (7)

**Table 3:** ABCD2 score and risk of stroke.

Risk category	ABCD2 3 or less (*N*)		ABCD2 4 or more (*N*)	
	Low risk score (*N*)		High risk score (*N*)	

	*N*	2-day risk % (*N*)	*N*	2-day risk % (*N*)

California, [37] 2005 (*N* = 3738)	562	2.49 (14)	3176	4.28 (136)
Oxford, [37] 2005 (*N* = 649)	247	.8 (2)	402	8.95 (36)

	*N*	7-day risk % (*N*)	*N*	7-day risk % (*N*)

California, [37] 2005 (*N* = 3738)	562	2.84 (16)	3176	5.88 (187)
Oxford, [37] 2005 (*N* = 649)	247	1.6 (4)	402	14.67 (59)
Tsivgoulis et al. [21] (*N* = 226)	84	1.19 (1)	142	11.97 (17)
Asimos et al., [25] 2009 (*N* = 1169)	NA	NA	NA	NA
Fothergill et al., [23] 2009 (*N* = 276)	68	5.9 (4)	208	15.4 (32)
Konton and Rothwell [35] (*n* = 278)	88	NA	190	NA

	*N*	30-day risk % (*N*)	*N*	30-day risk % (*N*)

Tsivgoulis et al., [21] (*N* = 226)	84	1.19 (1)	142	14.78 (21)
Fothergill et al., [23] 2009 (*N* = 276)	68	5.9 (4)	208	16.8 (35)

	*N*	90-day risk % (*N*)	*N*	90-day risk % (*N*)

California, [16] 2005 (*N* = 3738)	562	6.58 (37)	3176	9.6 (305)
Oxford, [16] 2005 (*N* = 649)	247	5.26 (13)	402	21.4 (86)
Josephson et al. [11] (*N* = 713)	190	6.31 (12)	523	26.95 (141)

**Table 4:** 

Population year location/type	*N* (Total Number)	Stroke risk (*n*)	% of all strokes in 2 days and 7 days out of 90 days
Johnston et al. 2000 [9]	1707	2-day risk = 83	2 days—46%
USA		7-day risk = 103	7 days—57%
ED based		90-day risk = 180	

Johnston et al. 2007 [16]	1069	2-day risk = 51	2 days—48%
USA		7-day risk = 71	7 days—67%
Outpatient		90-day risk = 106	

Johnston 2007 [16]	962	2-day risk = 16	2 days—29%
USA		7-day risk = 29	7 days—52%
ED based		90-day risk = 56	

OCSP 1986 [10]	203	2-day risk = 9	2 days—31%
Population		7-day risk = 17	7 days—59%
UK		90-day risk = 29	

OXVASC 2004 [10]	188	2-day risk = 13	2 days—39%
Population		7-day risk = 20	7 days—61%
UK		90-day risk = 33	

OXFORD 2005 [10]	315	2-day risk = 9	2 days—41%
Outpatient Clinic, UK		7-day risk = 17	7 days—77%
		90-day risk = 22	

Cucchiara et al. 2007 [38]	167	2-day risk = 4	2 days—80%
Dedicated Unit		7-day risk = 4	7 days—80%
USA		90-day risk = 5	

Tsivgoulis et al. 2004 [21]	226	2-day risk = na	2 days—na
ED		7-day risk = 18	7 days—78 %
Greece		90-day risk = 23	

Bray et al. 2004 [33]	98	2-day risk = na	2 days—na
ED		7-day risk = 4	7 days—57%
Australia		90-day risk = 7	

Calvet et al. 2007 [24]	343	2-day risk = 4	2 days—40%
Dedicated Unit		7-day risk = 5	7 days—50%
France		90-day risk = 10	

Purroy et al. 2005 [19]	204	2-day risk = 2	2 days—33%
ED		7-day risk = 3	7 days—50%
Spain		90-day risk = 6	

Asimos et al. 2008 [25]	1054	2-day risk = na	2 days—na
ED		7-day risk = 69	7 days—na
USA		90-day risk = na	

Fothergill et al. 1994 [23]	284	2-day risk = na	2 days—na
Population based		7-day risk = 36	7 days—na
USA		90-day risk = na	

Mlynash et al. [39] 2005	99	2-day risk = 0	2 days—0%
USA		7-day risk = 1	7 days—100%
Dedicated		90-day risk = 1	
Unit			

Ay et al. [40] 2006	586	2-day risk = 15	2 days—na
USA		7-day risk = 28	7 days—na
Dedicated Unit		90-day risk = na	

EXPRESS 2007	160	2-day risk = 1	2 days—100%
UK		7-day risk = 1	7 days—100%
Dedicated Unit		90-day risk = 1	

Intervention	1466		
SOS-TIA 2005		2-day risk = 2	2 days—12%
France		7-day risk = 5	7 days—29%
Dedicated Unit		90-day risk = 17	
Intervention			

Sciolla and Melis[20]	274	2-day risk = 7	2 days—47%
2006		7-day risk = 10	7 days—67%
Italy		90-day risk = 15	
ED			

Coutts et al. [41]	111	2-day risk = 2	2 days—33%
2006		7-day risk = 4	7 days—67%
Canada		90-day risk = 6	
ED	292		

Sheehan et al. [42]		2-day risk = 3	2 days—11%
2007		7-day risk = 11	7 days—39%
Ireland		90-day risk = 28	
Population		
